# Maternity and the Teaching Profession

**Published:** 1911-07-29

**Authors:** 


### MATERNITY AND THE TEACHING PROFESSION.

The conflict of interests which arises when a woman who earns her living in any kind of permanent employment has married and becomes pregnant, ends in almost every case in the abandonment of the profession and the victory of the claims of motherhood. It is quite proper that this should be so, for the rights of the infant are imperative; but in many cases those rights are recognised chiefly because the employer refuses to retain the services of the mother. There is one profession largely staffed by women in which maternity should be less of a bar to employment than in most others—that of teaching. But in point of fact the illiberality of school managers is so great that to become a mother is tantamount to losing all chance of employment; and even to marry is only too often equivalent to being compelled to resign. Yet the divisions of the school year and the vacations between the terms render it fairly easy for the services of a teacher to be dispensed with for four or five or six months, without prejudice to the efficiency of the school; long enough to allow the mother to nurse her child, at any rate, nearly up to the first dentition. Moreover, teaching is a profession for which normal gestation is really no grave detriment. Still, as we say, nearly all school authorities look askance on married (women) teachers. In these circumstances, the Ebbw Vale case, decided on July 15 in the King's Bench Division, is of distinct importance. The plaintiff, for many years in the employ of the Ebbw Vale Education Authority, was suspended when five months pregnant for that reason alone. She claimed full wages on the ground that she was not incapacitated, but was offered a gratuity of £10; this she refused to accept. In giving judgment, Mr. Justice Channell took the common-sense view that no sufficient proof of incapacity had been offered by the defence; no evidence was forthcoming to show that the teacher was in bad health; and certainly it cannot be contended that in the middle of a normal pregnancy an expecting mother is unfit to carry on her teaching. In the end a verdict for the full amount claimed was given.

With the wisdom and justice of this judgment we cordially agree; yet it is easy to see that it may in some ways do harm. It may make education authorities more than ever reluctant to employ married teachers and to allow the marriage of those in their employ. Further than that, it may mean that education authorities will be within their rights in dismissing not, indeed, those who are about to be mothers, but those who after their confinement spend a few months nursing their offspring; for the latter, one imagines, could quite reasonably be described as unfit or incapacitated from teaching. The decline of breast-feeding, and the toll which that decline takes of infant life and infant health, are terrible enough as it is without any additional inducements tending towards bottle feeding. It would be of great advantage if every education authority should include at least one medical man, preferably an experienced general practitioner, to instruct the other members in the technical aspect of questions such as that which arose at Ebbw Vale.

